# A Dynamic Systems Approach for Detecting and Localizing of Infarct-Related Artery in Acute Myocardial Infarction Using Compressed Paper-Based Electrocardiogram (ECG)

**DOI:** 10.3390/s20143975

**Published:** 2020-07-17

**Authors:** Trung Q. Le, Vibhuthi Chandra, Kahkashan Afrin, Sanjay Srivatsa, Satish Bukkapatnam

**Affiliations:** 1 Industrial and Manufacturing Engineering, North Dakota State University, Fargo, ND 58102, USA; 2 Industrial and Systems Engineering, Texas A&M University, College Station, TX 77843, USA; vib25889@gmail.com (V.C.); afrin@tamu.edu (K.A.); satish@tamu.edu (S.B.); 3 Heart Artery and Vein Center of Fresno, Fresno, CA 93722, USA; sanjaysrivatsa@hotmail.com

**Keywords:** electrocardiogram, nonlinear dynamic systems, computer-aided diagnosis

## Abstract

Timely evaluation and reperfusion have improved the myocardial salvage and the subsequent recovery rate of the patients hospitalized with acute myocardial infarction (MI). Long waiting time and time-consuming procedures of in-hospital diagnostic testing severely affect the timeliness. We present a Poincare pattern ensemble-based method with the consideration of multi-correlated non-stationary stochastic system dynamics to localize the infarct-related artery (IRA) in acute MI by fully harnessing information from paper-based Electrocardiogram (ECG). The vectorcardiogram (VCG) diagnostic features extracted from only 2.5-s long paper ECG recordings were used to hierarchically localize the IRA—not mere localization of the infarcted cardiac tissues—in acute MI. Paper ECG records and angiograms of 106 acute MI patients collected at the Heart Artery and Vein Center at Fresno California and the 12-lead ECG signals from the Physionet PTB online database were employed to validate the proposed approach. We reported the overall accuracies of 97.41% for healthy control (HC) vs. MI, 89.41 ± 9.89 for left and right culprit arteries vs. others, 88.2 ± 11.6 for left main arteries vs. right-coronary-ascending (RCA) and 93.67 ± 4.89 for left-anterior-descending (LAD) vs. left-circumflex (LCX). The IRA localization from paper ECG can be used to timely triage the patients with acute coronary syndromes to the percutaneous coronary intervention facilities.

## 1. Introduction

Rapid assessment and timely reperfusion therapy have shown to improve the recovery rate in patients with acute myocardial infarction (MI) [1]. The National Heart Attack Alert Program (NHAAP) guidelines recommend fibrinolysis and intervention within 30 and 90 min of an acute MI onset to maximize the restoration of the jeopardized myocardium [2]. However, according to Heart Disease and Stroke Statistics (2017) [3], only 50% of acute MI patients are treated with thrombolytic agents and 65% undergo percutaneous coronary intervention within the recommended golden hour at Veterans Health Administration’s (VHA) hospitals, the largest integrated health care system in the United States. The guidelines are often not followed in over-crowded hospitals due to the significant amount of delay between para-clinical first response to conclusive intervention [4]. Such a delay is incurred due to waiting and queuing at the overloaded emergency and the time consumed during various stages of elaborate lab diagnostic tests [5,6]. Many efforts have been devoted to reduce the door-to-device’s period lasting from the time the patient with ongoing ischemia receives the first response till the patient’s admission to the catheterization laboratory [4,6]. Accurate stratification of the coronary lesions and assessment of the infarct-related artery (IRA) in acute MI would significantly reduce such reperfusion delay.

Even though coronary angiography has been widely utilized for the localization of IRAs, prehospital 12-lead ECG print out has been highly recommended in ongoing ischemic patients supposed to be admitted to the revascularization facilities. Coronary angiography procedure uses a radio-opaque contrast agent and x-ray imaging via an arterial catheter to visualize the coronary arterial occlusions. Despite its accuracy, the radiation risk and invasive procedure hinder angiography from being the first choice of the doctors [7,8]. Its associated invasive procedure, time-consuming process and costly investment have prompted an intensive search for alternatives [8,9]. Recent non-invasive angiography technologies, such as electron beam angiography (EBA), computed tomography angiography (CTA) and magnetic resonance angiography (MRA), are the possible replacements for catheter-based angiography [10,11]. However, these methods are constrained by low temporal-resolution, high noise susceptibility and capital-intensive equipment [11,12]. American Heart Association national guidelines [13] and other consensus and scientific statements [14] have recommended the use of prehospital 12-lead ECG paper printout that can be rapidly recorded using portable equipment on first responder’s emergency vehicles to evaluate the patients with suspected acute coronary syndrome (ACS) to decrease delays in reperfusion therapy. Despite that 90.6% of emergency medical services (EMS) serving the 200 largest US cities are equipped with 12-lead ECG in the ambulance system, prehospital ECG has been only used in 15% of patients with acute MI [15]. Thus, even though the equipment is available, it is mostly underutilized. One of the challenges is the derivation of statistically consistent features from the reconstructed VCG using paper ECG that can provide direct confirmation of MI to urgently facilitate the activation of the reperfusion facilities [16].

Computer-aided diagnostic methods have been proposed to detect and localize MI, yet very few works focus on using ECG readings to localize the infarcted cardiac artery. Automated diagnostic methods focus on novel features extraction techniques or machine learning algorithms to detect and classify MI cases. Arif et al. [17] used T-wave inversion, Q-wave and ST-level elevation, reporting accuracy of 98.3% on ten classes of MI with a KNN classifier. Sadao and Senya [18] utilized purpose-oriented feature extraction to separate three classes of myocardial and normal subjects with the reported sensitivity of 86% for normal, 93% for anterior, 80% for inferior and 93% for flat T wave MI. O’Dwyer et al. [19] used several feature sets including wavelet-based and standard morphological ECG features to detect various types of MI with the overall accuracy ranged between 60% and 75%. Le et al.’s [16] approach to localize MI based on representing the complex spatio-temporal patterns of cardiac dynamics as a random-walk report the sensitivity of 88% and specificity of 92%. Strodthoff et al. [20] utilized a deep neural network detecting the MI from ECG signals with the sensitivity and specificity of 93.3% and 89.7%, respectively. Despite the efficacy in classification and localization of MI using ECG related features, very few of the previous works’ methods focus on localizing the culprit infarct-related arteries (IRAs), which primarily cause the acute MI.

Towards addressing the challenges in identifying IRAs using paper ECG, we developed a method to reconstruct the diagnostic-quality vectorcardiogram (VCG) signals from paper ECG recordings to localize the IRA of acute MI. Several challenges need to be addressed for the utilization of paper ECG to localize the IRA in acute MI. Due to the selected representative display and uneven resolution of the waveforms on the paper ECG, the diagnostic ECG’s intervals are usually underestimated. In addition, the waveform amplitudes in the simultaneous lead presentation of the paper ECG are systematically greater than those in the corresponding measurements made from the single lead [21]. Furthermore, the 2.5-s ECG signals from the paper printouts were not sufficient enough to obtain statistically consistent estimates for the machine learning algorithm to localize IRA [16]. These diagnostic errors hinder the use of paper-based ECG signals for the precise localization of the IRA [21,22]. We proposed a nonlinear dynamic signal reconstruction method using Poincare sectioning to generate statistically consistent features from paper ECG for the MI localization. A Poincare pattern ensemble reconstruction method based upon our previous work [23] on the Karhunen–Loeve (KL) representation of the ECG signal was proposed to estimate the missing heartbeats and subsequently reconstruct the full 10-s length of the 12-leads ECG. The missing ECG signals were derived from the KL basis functions with the accurate time alignment from the 10-s ECG lead. The full 10-s length of 12-lead ECG signals constructed using Poincare pattern ensemble reconstruction method provided statistically consistent VCG features to classify various infarcted related arteries including left anterior descending (LAD), left circumflex (LCX), right coronary ascending (RCA) and exact location not discernible (E). In the absence of the signal’s realization, this reconstruction can account for the nonlinear and stochastic dynamic characterizations attributed to the complex behaviors of the underlying cardiac system [23,24]. This method outperformed the simultaneous lead format in augmenting the time-coherent data and reconstructing representative template complexes which are critical to feature extraction for the localization of IRA.

The organization of this paper is as follows: Section 2 describes the method to derive the diagnostic ECG and VCG from the paper recordings and the model to hierarchically localize the IRA in acute MI, Section 3 reports the results on the validation of ECG signal reconstruction, feature extraction and hierarchical classification of IRA and Section 4 and Section 5 discuss the findings and conclude the proposed method.

## 2. Methods

**Figure 1:**
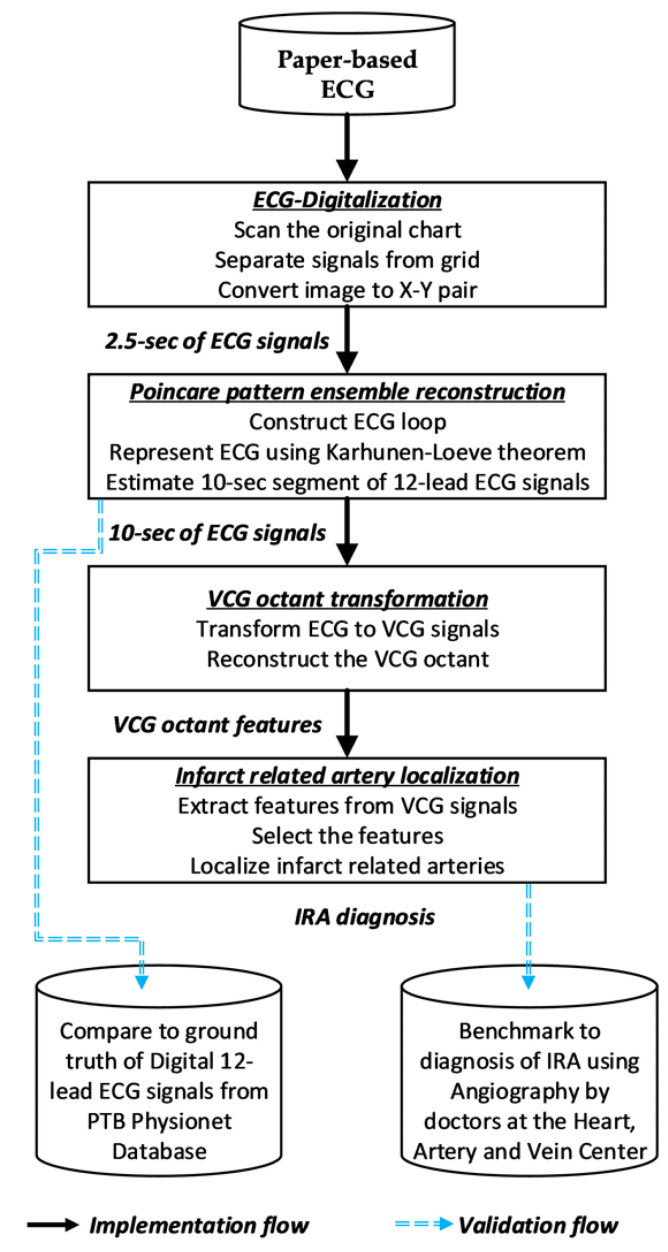
Summary of the proposed method to reconstruct the ECG diagnostic signals and localize IRA from the paper ECG.

The key contributions of the present work are in (1) a Poincare sectioning ensembles method to reconstruct missing ECG signals from the compressed paper recording that can preserve the multi-correlated non-stationary stochastic characteristics of the missing signals and (2) a computational framework to using angiographical-based hierarchical classification model with nonlinear features from the reconstructed signal to localize IRA. As summarized in Figure 1, we utilize a full strip of a paper ECG to scan for the 2.5-s segment of the 12-lead ECG. A 10-s segment of each lead is reconstructed using Poincare pattern ensemble reconstruction method. The ECG signals were transformed into vectorcardiogram (VCG) signals from which the spatial and temporal characteristics of the electric heart vector are extracted. A customized affine transformation that accounts for myocardial infarction condition has been utilized for the ECG-VCG transformation [25]. Finally, hierarchical classifiers are developed to map the extracted features into different IRA types determined by the occlusive coronary arteries. The validations are to compare the reconstructed ECG using ground truth from the 12-lead ECG from PTB Physionet Database [26] and to evaluate the accuracy of the model with the benchmarks to the IRA using coronary angiography of MI patient from Heart, Artery and Vein center in Fresno, California.

### 2.1. ECG Digitalization

**Figure 2:**
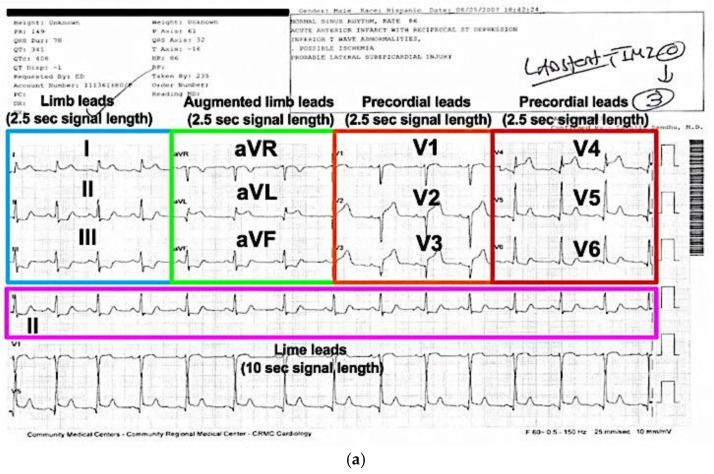

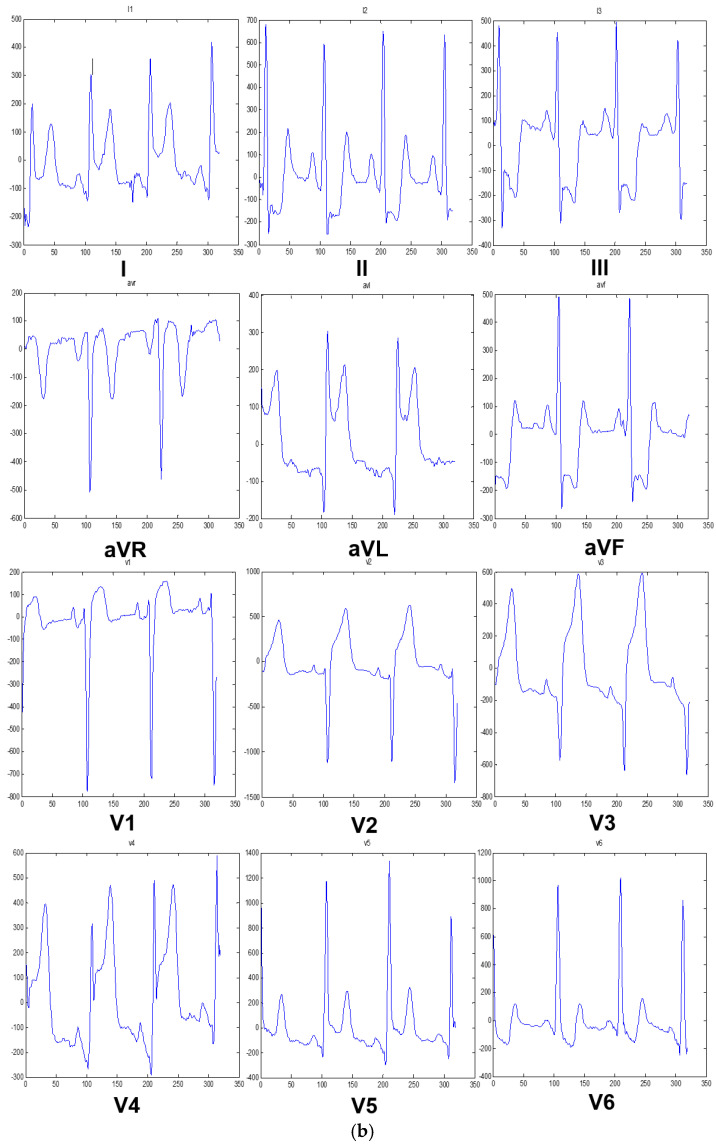
(**a**) Image of a paper ECG with 2 s long signal of a representative patient showing segments of limb (I, II and III), augmented limb (aVR, aVL and aVF) and precordial (V1-6) leads and a full 10 s of lead II signal. (**b**) The digitized waveform of the 12-lead ECG signals with the *X*-axis represent the time duration (ms) and the *Y*-axis represent the signal amplitude (mV).

The images of the ECG signals on the printout paper was initially digitalized. According to the American Heart Association’s standardization, the most common form of paper ECG is the simultaneous lead presentation, in which four 2.5-s columns are presented sequentially on the page with no time disruption between the columns. Each column, therefore, represents successive 2.5-s intervals of a continuous 10-s record from the limb, augmented limb and precordial leads (as shown in Figure 2). Specifically, the first column records three rows representing simultaneous three limb leads of I, II and III; the second column representing augmented limb leads of aVR, aVL and aVF; the third column represents precordial leads of V1, V2 and V3; and the fourth column represents simultaneous precordial leads of V4, V5 and V6. Additional rows may be available for 1-, 2- or 3-leads of 10-s continuous recordings for rhythm analysis.

In the digitalization step, the grids of the recording were initially removed by the grayscale thresholding techniques and the signals were separated as the black pixels from the background at the resolution of 144 dpi by the greyscale thresholding and pixel by the vector conversion method [27]. The coordinates of three points (0,0), (0,1000), and (200,0) were selected which corresponding to the signal amplitude of 1 mV and the signal duration of 200 ms. The bounds of the red, green, and blue threshold to separate the waveforms and the background were selected manually by sampling the color along the signal curves. The black pixels, representing for the waveforms, were extracted and concatenated in the column-wise form to generate the digital signals. As a result of the digitization, a full 10-s digitized signal from lead II of the ECG, and 2.5-s signal of 12 channels I, II and III (limb leads); aVR, aVL and aVF (augmented limb leads); and V1, V2, V3, V4, V5 and V6 (precordial leads) were collected. A representative sample of a paper-based record and its digitized form of a 12-lead ECG are shown in Figure 2a,b. The WinDigData digitizer software was utilized to assist the digitization process and the detailed steps can be found from the Digitization Workflow site [28].

### 2.2. Poincare Ensemble Reconstruction and VCG Transformation

**Figure 3:**
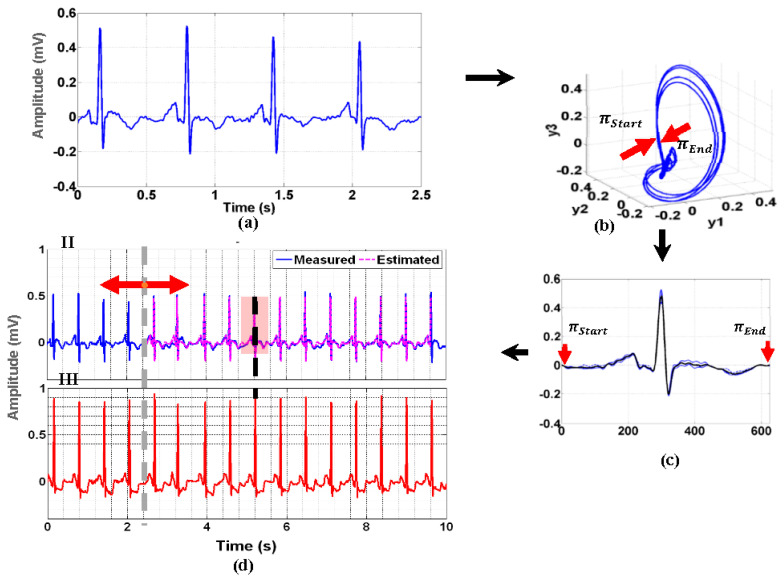
Summarized steps (**a**–**d**) for the reconstruction of 10-s of lead II using 2.5-s of lead II and 10-s of lead III in a representative MI patient using Poincare pattern ensemble method.

Poincare sectioning ensembles of the ECG phase space trajectory were proposed to reconstruct the representative template of the ECG signal. Since the 2.5-s signals from the digitized 12-lead ECG were too short to obtain statistically consistent estimates for the IRA localization, a Poincare pattern ensemble approach was developed to reconstruct a full 10 s of 12-leads ECG. Figure 3 illustrates the ECG reconstruction for one lead of the digitized ECG signal. The nonlinear dynamic state space of the underlying cardiac system was initially constructed from a 2.5-s segment (Figure 3a) of each digitized ECG channels using Taken’s embedding theorem [29]. The time evolution of the state space’s trajectories (Figure 3b) explains the nonlinear dynamics of the underlying cardiac system [30,31]. In the reconstructed state space, the collective points from $\pi_{Start}$ to $\pi_{End}$—the intersections of a ($d_{E}-1$) dimensional hyperplane also called the Poincare section and the signal trajectory—depict the returning map (Figure 3c). Here, the segments between successive $\pi_{Start}$ and $\pi_{End}$ in the returning map are called sectioning ensembles which variations characterize for the heart rate variability dynamics and $d_{E}$ is the embedding dimension determined by the Taken’s embedding theorem. These Poincare sectioning ensembles of the ECG phase space trajectory are near periodic which can effectively be utilized for the Karhunen–Loeve (KL) representation of the ECG signal [23,32].

The KL eigenfunctions estimated from the covariance matrix of the Poincare ensembles were utilized as the basis functions to estimate the missing heartbeats and subsequent full 10-s length of the missing leads. The KL spectral representation of a heartbeat of ECG signal is given by $g\left(t\right)=\overset{N}{\underset{i=1}{\sum }}\alpha_{i}\phi_{i}\left(t\right)$ with $\alpha_{i}$ is the KL coefficients and $\phi_{i}\left(t\right)$ is the linear independent solution of the function $\int_{0}^{T_{e}}K(t,T)\phi_{j}\left(T\right)dT=\vartheta_{j}\phi_{j}\left(t\right).$ Here $\phi_{i}\left(t\right)$ are eigenfunctions and $\vartheta_{j}\;$ are the eigenvalues of the covariance matrix K(t,T)  calculated from $T_{e}\;$ Poincare section ensembles. N is the number of dominant eigenfunctions explaining more than 95% percent of the total variance and $\alpha_{i}$ are the KL coefficients estimated from the same beat in the 10-s continuous recordings (Lead II in this paper). Poincare section ensembles were estimated simultaneously from $\pi_{Start}$-$\pi_{End}$ cut from 2.5-s signal realization as shown in Figure 3a–c. Based on the KL spectral expansion, the 10-s ECG segment was estimated from the ensemble sets derived from the 2.5-s signal segment such that the 7.5-s missing part was interpolated from the KL representation of the heart beat g(t) with the R-peak aligned with the corresponding R-peak of the full 10-s ECG lead (Figure 3d). The steps were applied to the other ECG leads to reconstruct a full set of 10-s of 12-leads of ECG. Eventually, the reconstructed 12-lead ECG was transformed into 3-lead VCG by using an empirical transformation matrix [25]. The spatial-temporal dynamics and stochastic variations of the cardiac dipole vector were quantified by the Octant and Network random-walk features extracted from the VCG [23,25].

In the digitalization step, the grids of the recording were initially removed by the grayscale thresholding techniques and the signals were separated as the black pixels from the background at the resolution of 144 dpi by and pixel by the vector conversion method [27]. The black pixels, representing for the waveforms, were extracted and concatenated in the column-wise form to generate the digital signals. As a result of the digitization, a full 10-s digitized signal from Lead II of the ECG, and 2.5-s signal of 12 channels including I, II and III (limb leads); aVR, aVL and aVF (augmented limb leads); and V1, V2, V3, V4, V5 and V6 (precordial leads) were collected. A representative sample of a paper-based record and its digitized form of a 12-lead ECG are shown in Figure 2. The WinDigData digitizer software was utilized to assist the digitization process.

### 2.3. Feature Engineering and Infarct-Related Artery (IRA) Localization

**Figure 4:**
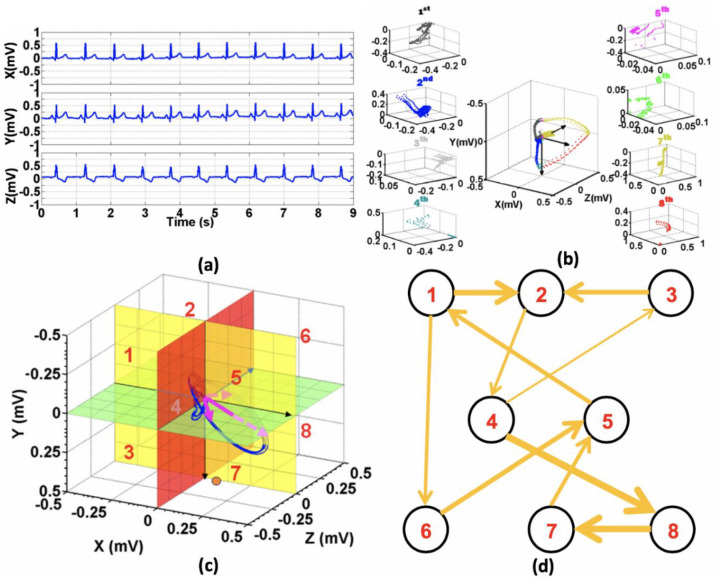
Steps to formulate the directed weighted network of a cardiac vector in the octant space from a vectorcardiogram signal from a representative subject; (**a**) time-portrait of three VCG channels X, Y and Y; (**b**) VCG trajectory in a 3D Cartesian octant space and 8 octants with the signal realizations in each octant are color-coded; (**c**) temporal transitions of VCG trajectory among 8 octants defined by the 3D Cartesian octant space; and (**d**) directed weighted network with the nodes are 8 octants and the weighted edges are the transitions of VCG trajectory among the octants.

Four groups of features were extracted from the reconstructed VCG octants with the detailed processing steps presented in our previous work [16]. In particular, the method involves representing the evolution of VCG signals as a dynamic network in a 3D state space and characterizing the random-walk transition of the cardiac vector in the network to track the spatio-temporal dynamics of the underlying cardiac system. Figure 4 summarizes the overall steps to model a directed weighted network of the cardiac vector with the nodes and edges defined from VCG cartesian octant space. Four feature groups extracted from the octant network consist of: (1) local octant features, (2) octant residence features, (3) octant transition features and (4) network topology features. The local octant group includes 48 descriptive features, such as the minimum, average, variance, maximum, azimuth and elevation of the vector magnitudes. These features are attributed to the morphological and temporal characteristics of the VCG signals. The octant residence group comprises 12 features which include sojourn times in each octant and velocity of the vector magnitudes. The octant transition group consists of 16 features including arrival and departure rates between each pair of octants. Finally, 85 features from network topology quantify the topology of the random-walk network, including the degree of assortativity (48), density clustering (17), distances and cycles (12) and betweenness centrality (8). A summary of these features is described in Table 1.

Principal component analysis (PCA) and bootstrapping were used to reduce the dimension of the feature space and address the imbalance of samples among different IRA groups in the dataset. The principal components (PCs) explaining 80% of the feature’s variance were selected for the representation of the feature set. Due to a large number of features from the octant analysis, a subset of significant features was selected from the feature space based on its contribution to selected PCs. The contribution of the feature $k^{th}$ to the PC is defined as $w_{k}=\overset{n}{\underset{i=1}{\sum }}\beta_{i}*c_{ki}^{2},$ where $\beta_{i\;}$ is the eigenvalue of the principal component i and $c_{ki}$ is the loading of the feature $k^{th}$ on the principal component i. The features with the highest contributions were selected as the feature set for the classification of different infarct-related arteries. Borderline-Synthetic Minority Over-sampling Technique (SMOTE) [33]—an over-sampling method in imbalanced dataset learning—was performed to create a balanced training set to establish a stable decision boundary.

**Figure 5:**
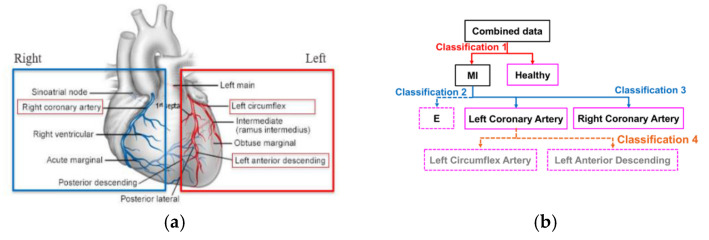
(**a**) Anatomy of the heart with the highlighted left and right coronary arteries (http://medchiefs.bsd.uchicago.edu/); and (**b**) hierarchical classification tree with four classifiers to localize different culprit arteries. Classifier 1 separates healthy subjects and the MI patients. Classifier 2 distinguishes the MI cases with discernable infarcted locations from those with indiscernible locations. Classifier 3 splits the culprit arteries in right and left coronary arteries. Classifier 4 determines the infarction in left circumflex and left anterior descending arteries in the left main branches.

We proposed an angiographical-based hierarchical classification model on the selected features to classify different IRA. The hierarchy of the classifier assimilates the catheter-based angiography procedure that traces the occlusion along the branches of infarct-related arteries. This top-down strategy utilizes pre-defined classes determined by the hierarchy of the coronary anatomy as the predicted classes to minimize the misclassification errors in a multiclass classification problem [34]. Figure 5a illustrates the anatomical structure of the heart with the highlighted main coronary arteries: left coronary artery (red) and right coronary artery (blue). Based on the anatomical arrangement of the major arteries, a hierarchical structure of the classification models to stratify various IRA groups was proposed, as shown in Figure 5b. At the first level, classification is sought between the healthy subjects and the MI patients. On the MI patients, the model resume to classify the occlusions on the left or right branches. If the occlusions are on the left branches, the model specifically discerns the occluded locations on the left circumflex artery (LCA) or left anterior descending (LAD) artery. The hierarchical implementation of this model was similar to the sequence of the angiography catheter being maneuvered through the coronary arteries to identify the culprit IRA in acute MI.

## 3. Implementations and Results

### 3.1. Data Descriptions

Two sources of data were employed for the validation of the presented method. The first source of data consisted of 362 recordings (282 MI and 80 HC) from the PhysioNet PTB Database. Each recording contained 15 simultaneous channels of signal, namely, the conventional 12-lead ECG and the 3-lead (Frank XYZ) VCG. The signals were sampled at 1 kHz with a 16-bit resolution over a range of ±16.384 mV for at least 30 s with an average length of ∼2 min. This first source of data was used to evaluate the consistency of the reconstructed ECG from the paper ECG and the ground truth from the 12-lead ECG. The second source of data was the paper ECG printouts and the coronary angiography collected from the patients with the clinical symptoms of acute coronary syndrome presented to the Heart, Artery and Vein Center at Fresno California [35]. The ECGs were obtained at the paper speed of 25 mm, gain of 10 mV and paper format of 3 × 4 using a Philips Pagewriter Touch Interpretive ECG machine (Philips Medical Systems, Andover, MA, USA). Among the 171 patients who underwent a 12-lead ECG, 65 patients were excluded due to the poor quality of the collected signals. The excluded ECG data had low signal-to-noise ratio and artifact caused by the poor skin-to-electrode connection or mechanical factors such as equipment malfunctions. Nonetheless, in most scenarios, nurses are trained to identify the sources of artifacts and can repeat to get better measurements [36]. Among the remaining patients, those with acute MI were diagnosed by ECG tracings and confirmed by cardiac biomarkers. Subsequently, coronary angiography was performed to classify the acute MI cases into four groups based on the localization of the infarct-related arteries. The four culprit artery groups were annotated as (i) LAD (left anterior descending), (ii) LCX (left circumflex), (iii) RCA (right coronary artery), (d) E (for those patients whose MI locations are not clearly identified). The second data source was used to cross-validate the accuracy of the IRA classification model. Table 2 summarizes the demographics of the selected study cohort in the second data source.

### 3.2. Reconstruction of 12-Lead ECG Signal

The 12-lead ECG measurements collected from the online Physionet PTB database have been used to validate the Poincare pattern ensemble reconstruction approach. Accordingly, we formulated the digitized signals of paper ECG by getting 10 s of lead II ECG and 2.5 s of other leads from the full 12-lead ECG. The Poincare pattern ensemble approach was applied to the formulated signals to reconstruct the full 10-s of 12-leads of ECG. The reconstructed signals were finally compared with the full 12-lead ECG to evaluate the performance of the reconstruction method. The correlation coefficients R^2^—goodness of fit between the estimated and the measured signals—of the estimated ECGs and the measurements from 12-lead ECG on the PhysioNet PTB Database are reported in Table 3. The R^2^ statistic of 1 indicates that reconstruction is able to correctly reproduce the actual measured data every single time. The $R^{2}$ statistic in lead i is given by:

(1) $$R^{2}=1-\frac{\sum_{Signal\;length\;in\;lead\;i\;}{\left[Reconstructed\left(sample\;k\right)-Measured\left(sample\;k\right)\right]}^{2}}{\sum_{Signal\;length\;in\;lead\;i\;}{\left[Measured\left(sample\;k\right)\right]}^{2}\;}$$

**Figure 6:**
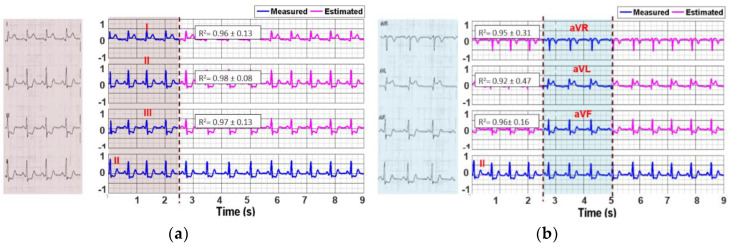

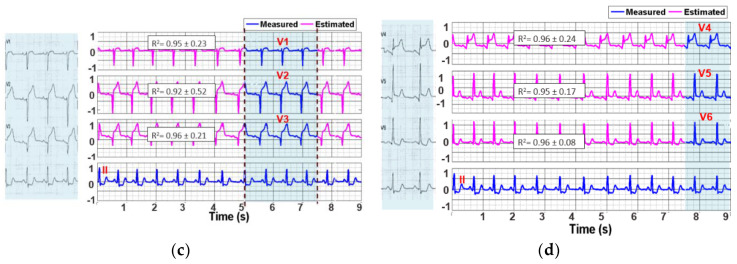
A representative 10 s long ECG signal of an MI patient reconstructed from 2.5-s paper-based signals collected from (**a**) limb leads, (**b**) augmented limb leads and (**c**,**d**) precordial leads.

The reported R^2^ in 12-lead ECG are above 0.95, indicating a highly accurate estimation of full 10-s long ECG from the 2.5-s signals from the paper recordings. The algorithm was performed on the digitalized ECG signal for the full 12-lead of the paper-ECG. Figure 6 demonstrates a representative record of a 2.5-s segment of the paper ECG signal, the measured and the estimated signals of the 12-lead ECG in (a) limb leads I, II and III; (b) augmented limb leads aVR, aVL and aVF; (c) precordial leads V4, V5 and V6; and (d) precordial leads V1, V2 and V3.

### 3.3. Feature Extraction and Feature Selection

**Figure 7:**
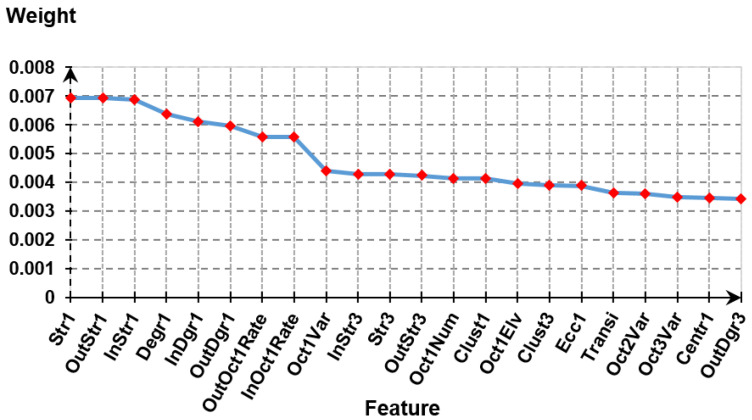
A set of 22 features and their corresponding weight values selected from the PCA analysis with the cut-off at the feature OutDgr3.

PCA was performed on the extracted feature space to justify the redundancy of the extracted features. Of all the 151 features extracted from the reconstructed signals, 22 features were selected for further analysis. Figure 7 shows 22 selected features with their corresponding weight value w in descending order. The total weights accounting for 80% of the variation of the feature space with the cut-off at OutDgr3 feature was used to determine the threshold to select the features. This feature set was used to separate the healthy subject and MI patient groups.

**Figure 8:**
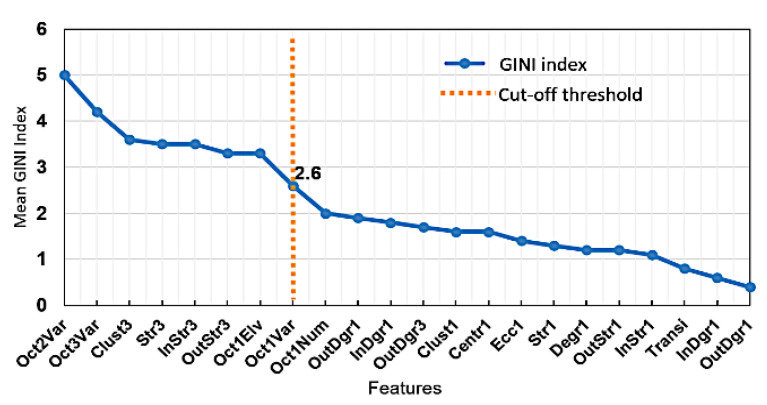
Variable importance plot of the selected features with the cut off threshold specified.

To avoid the overfitting issue and increase the interpretability of the hierarchical model, a subset of the 22 above features was selected based on PCA analysis for the IRA localization. Figure 8 indicates the variable importance plot of Gini’s diversity index, a statistic to represent the reduction in the accuracy of the classification model in the absence of a specific feature. It is observed from the figure that Oct2Var was the most significant feature and Oct1Var was the cut-off feature to separate the feature space into two separate groups. The cut-off threshold is determined based on the significant reduction of the Gini’s diversity index. Thus, the reduced feature set consisting of the first eight features, including Oct1Var, was selected as the classifier inputs to separate different infarct-related arteries. In the collected dataset, the number of samples under different classes was unbalanced as follows: E-25, LAD-42, LCX-8, RCA-31 and HC-80 cases. Accordingly, due to the imbalance between the IRA training classes, more weight would be given to the samples nearer to the classifier boundary, and thus the classification model decision would be biased to the groups with more observations. We performed the borderline-SMOTE over-sampling method to generate minority class instances on the line segments joining each minority class instances with its nearest neighbors.

### 3.4. IRA Localization and Hierarchical Model Selection

**Figure 9:**
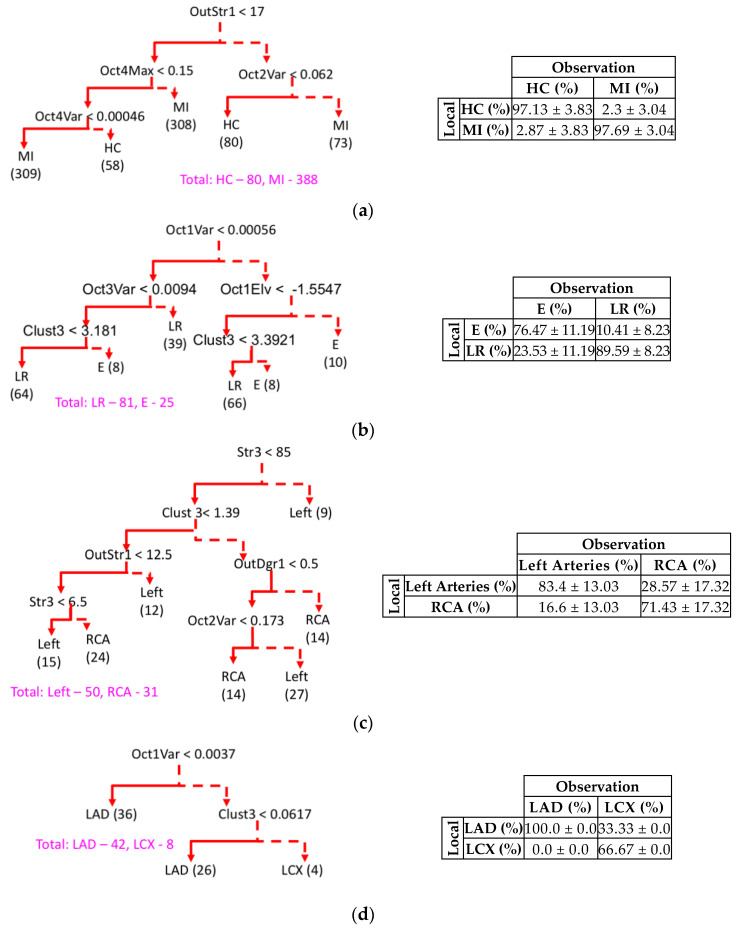
Summary of CART trees and corresponding contingency matrix of various IRA localization. All CART models are specified in terms of a trees structure with the solid lines denoting the true branch (i.e., the condition stated at the root of the tree holds) and the dashed line denoting the false branch. The optimized model structures are showed in (**a**–**d**). At the first level, classification was sought between healthy subjects and MI patients (**a**); later, the culprit arteries were classified into in left and right arteries (LR) vs. unspecified (E) in (**b**); and the specified occlusion was localized on left main arteries or right arteries (RCA) in (**c**). If it was on the left, the model continues to determine whether it is left circumflex artery (LCX) or left anterior descending (LAD) branches (**d**).

The results of the classification for different IRA types were reported. The 10-fold validation with the repeated application of bootstrapping data was performed to handle the data unbalance and avoid the overfitting problem. The occlusions in three major coronary arteries to supply blood to the myocardium, including LAD (50%), RCA (25%) and LCX (25%), were investigated. From Figure 9a, the classification tree detected HC and MI with the specificity of 97.69% and the sensitivity of 97.13%, respectively. The octant features in group I and network features in group IV, including OctVar, OctMax and OutStr, were selected as significant ones for the separation between MI and HC. Figure 9b shows the decision tree to separate between E and Left-Right (LR) classes with the selected features from the network feature (group IV) of Clust and three octant features (group I) of “Oct1Var”, “Oct3Var” and “Oct1Elv”. An accuracy of 89.59% and 76.47% were reported for the group LR and E, respectively. The lower accuracy of group E was attributed to the indiscernibility of this group from the LAD, LCX and RCA groups. Four network features, including “Str3”, “Clust3”, “OutStr1” and “OutDgr1”, were significant features in detecting the left main arteries and RCA group as shown in Figure 9c. As opposed to the inability of electrocardiographic features in classifying left and RCA classes [16,25], the selected features yielded the accuracies of 83.4% and 71.43% on these two classes, respectively. The overlapping of the LCX with the RCA could be the reason for the misclassified cases of the left and the right arteries. In the last CART as shown in Figure 9d, the model over classifies the LAD with the accuracy of 100%, while the accuracy of LCX was reported as 66.67%. Such misclassification of LAD over LCX results from the dominance of the LAD’s branches over the LCX’s branches.

A group of classification models was reported for the optimal classification model selection. The candidate models used to implement the IRA classification include the classification regression tree (CART), support vector machine (SVM), k-nearest neighbor (KNN), neural network (NN) and bagged ensemble tree (BET). The cross-validation was performed to improve the generalization of the model and the parameters of each model were selected optimally from the training and validation process. Table 4 shows the average classification results at all hierarchical levels using CART, SVM, KNN, NN and BET. It is noted that the classification accuracies for different IRA classes were consistent, which indicates the robustness of the selected feature set. Among the classification models, CART excelled in detecting MI condition, with an average accuracy of 97.41%; meanwhile, SVM was superior in specifying different infarct-related arteries, with the average accuracy >88%. Among the reported accuracy, the classification of LAD vs. LCX yielded the best overall results of 93.67% by using SVM; however, the classification of RL vs. E produced the equivalent results by using SVM and NN. Based on the classification accuracy, we proposed the use of the CART hierarchical classification as the optimal model for the detection of MI vs. HC and SVM for the localization of different IRAs.

## 4. Discussion

The interpretation of the octant features and the location of the occlusive coronary arteries are discussed. Since in MI cases, the mean electrical axis of depolarization deviates from the normal range by 20–90 degrees [37], most of the octant transitions characterized by ischemia changes shift from the left side in octants 5–8 to the right side in octants 1–4, as shown in Figure 4c,d) and in our previous work [16]. As a result, most octant features in octants 1–4 were selected for the separation between healthy from MI as well as coronary arterial occlusions. The vector transition shifting among octants was manifested by the T-wave inversion (the sign of ischemia and infarction evolution), ST segment (the indication of an on-going injury) and the known characteristics of Q-wave (manifestation of long-term necrosis) in traditional 12-lead ECG. The loss of positivity in the infarcted areas after the depolarization is responsible for these vector transition shifting. In particular, for the classification of MI from normal cases, features from octants 1, 2 and 4 were used. The vector amplitude was also higher in octant 4 (Oct4Max), due to the inverted T-wave resulting in MI cases. More transitions to octant 3 imply more activities on the right side due to the ischemia areas on the left side. If there are fewer activities around octant 3 (which is indicated by low clustering coefficient), then more activities in octant 1 show the indication of occlusion in the right arteries.

## 5. Conclusions

One of the most important clinical questions after an acute MI is the culprit arteries and the inducible extension of the ischemic myocardium. The timely confirmation of the ischemic locations and the IRA in an acute MI allows the patient to be directed to the catheterization laboratory, even bypassing the emergency department. In this paper, we developed a method to estimate the missing heartbeats and reconstruct the full 10-s length of the 12-leads ECG for timely classification and localization of the culprit infarct-related arteries. The proposed method was to reconstruct the VCG signals from the paper ECG, which can preserve the dynamic characteristics of the missing signals, as well as a computational framework to utilize paper-based ECG records for the detection and localization of IRA. Taken’s theorem-based reconstruction method, using the Poincare session on the state space, was proposed to extract fiducial patterns and recurrent structures of the signal pattern without losing the dynamic characteristics of the original ECG signals. The KL eigenfunctions estimated from the covariance matrix of the Poincare ensembles were utilized as the basis functions to estimate the missing heartbeats. By using the Poincare pattern ensemble reconstruction method, a full length of 10-s of 12-lead ECG signals was constructed from a 2.5-s long signal of the paper ECG recordings. This full length of 10-s ECG signal and its derived VCG signals provided asymptotic consistent estimators for a machine learning localization model. The reconstructed ECG signals were found to be highly correlated (R^2^ > 92%) to the benchmark 12-lead ECG signals collected from the online Physionet database. A hierarchical classification model, mimicking a catheter-based angiographic procedure, was developed for the localization of the IRA. The features extracted from the constructed signals were employed as the input of the classification model to determine different IRAs with an accuracy of 97.41% for healthy control (HC) vs. MI, 83.03% for E vs. others, 77.42% for LAD and LCX vs. RCA and 83.34% for LAD vs. LCX. One of the limitations of the model is the ability to localize multiple culprit arteries present in approximately 1% of angioscopy studies of acute coronary syndromes. Overfitting of rare IRA cases, especially LCX and E, can be addressed by analyzing larger retrospective pooled data with appropriate bootstrapping techniques. Our future research direction is to gain access to a larger dataset so that multiple IRAs can be simultaneously diagnosed. The proposed computational framework can be helpful to obviate critical complications and guide MI patient management at overcrowded emergency departments. The proposed method will enhance the development of advanced and cost-effective solutions for clinical diagnosis and cardiac care to the vast majority of healthcare settings across the world where capital investment to upgrade the current paper-ECG cardiac monitoring system to the digital Holter ECG has been limited.

## Figures and Tables

**Table 1:** Description of octant features extracted from the VCG signals.

Feature Groups	Feature Name (No. of Features)	Description
Local Octant (I)	OctiMin (8)	Minimal vector magnitude in octant i
	OctiAvg (8)	Average vector magnitude in octant i
	OctiVar (8)	Vector magnitude variance in octant i
	Octi1Max (8)	Amplitude of the maximal vector in octant i
	Octi1Elv (8)	Elevation of the maximal vector in octant i
	OctiAzm (8)	Azimuth of the maximal vector in octant i
Octant Residence (II)	OctiNum (8)	Sojourn time of the vector in octant i
	SlowTran	Minimal of octant transition rate in 10 s
	FastTran	Maximal of octant transition rate in 10 s
	MeanTran	Average of octant transition rate in 10 s
	VarTran	Variation of octant transition rate in 10 s
Octant Transition (III)	InOctiRate (8)	Arrival rate to octant i from all other octants
	OutOctiRate (8)	Departure rate from octant i to all other octants
Octant Network Topology (IV)	InDgri (8)	Number of inward links to octant i
	OutDgri (8)	Number of outward links from octant i
	Degri (8)	Octant i node degree
	InStri (8)	Sum of inward link weights to octant i
	OutStri (8)	Sum of outward link weights from octant i
	Stri (8)	Octant i node strength
	Clusti (8)	Clustering coefficient of octant i
	Jod	Number of octant with outward links > inward links
	Jid	Number of octant with inward links > outward links
	Jbl	Number of octant with inward links = outward links
	Rass	Assortativity coefficient of the octant network
	Kden	Number of octants with transitions
	Nden	Number of connections in the network
	K_den	Density of the octant network
	Transi	Transitivity coefficient of the network
	Qmod	Maximized modularity coefficient
	LambdaNet	Average shortest path length in the octant network
	EfficiencyNet	Average inverse shortest path length (Global efficiency)
	Ecci (8)	Greatest of all shortest path from octant i to all other octants
	RadiusNet	Radius of the octant network
	DiameterNet	Diameter of the octant network
	NodeBeti (8)	Node betweenness centrality of octant i

**Table 2:** Baseline characteristics of MI subjects admitted to the Heart, Artery and Vein Center at Fresno California.

Characteristics	Value	Characteristics	Value
Ethnicity	Hispanic 32.1%	Systolic Blood Pressure (mmHg)	121 ± 26.59
	Asian 5.7%		
	Caucasian 21.7%		
	Black 7.5%		
	Eastern Indian 2.8%		
	Unknown 30.2%		
Gender	Male 66.9%	Diastolic Blood Pressure (mmHg)	71 ± 16.26
	Female 33.1%		
Age	40.02 ± 14.08	Cholesterol	167 ± 51.38
Weight (lbs)	175.58 ± 50.02	BMI	28.39 ± 6.26

**Table 3:** Correlation coefficients between the reconstructed ECG and the measurements in 12-leads of ECG signal.

Lead	R^2^ Value (Mean ± Std.)	Lead	R^2^ Value (Mean ± Std.)
I	0.96 ± 0.13	V1	0.95 ± 0.23
II	0.98 ± 0.08	V2	0.92 ± 0.52
III	0.97 ± 0.13	V3	0.96 ± 0.21
aVR	0.95 ± 0.31	V4	0.96 ± 0.24
aVL	0.92 ± 0.47	V5	0.95 ± 0.17
aVF	0.96 ± 0.16	V6	0.96 ± 0.08

**Table 4:** Classification accuracies of different classes using CART and SVM models.

Models	Level in the Hierarchy Model			
	HC vs. MI	LR vs. E	LCA vs. RCA	LAD vs. LCX
CART	97.41 ± 2.44	83.03 ± 9.71	77.42 ± 5.18	83.34 ± 0.0
SVM	91.07 ± 4.46	89.41 ± 9.89	88.2 ± 11.6	93.67 ± 4.89
KNN	88.4 ± 0.75	84.57 ± 2.96	84.58 ± 3.52	88.89 ± 1.65
NN	85.75 ± 2.89	89.5 ± 4.37	76.13 ± 15.08	87.57 ± 6.92
BET	91.3 ± 0.69	77.73 ± 2.66	64.01 ± 6.59	84.49 ± 3.88
